# Individual-level modifiers of the acute effects of air pollution on mortality in Wuhan, China

**DOI:** 10.1186/s41256-018-0080-0

**Published:** 2018-09-06

**Authors:** Peirong Zhong, Shichun Huang, Xiaotong Zhang, Simin Wu, Yaohui Zhu, Yang Li, Lu Ma

**Affiliations:** 1 Department of Healthcare Management, School of Health Sciences, Wuhan University, 115 Donghu Road, Wuchang District, Wuhan, 430071 China; 2 Hubei Provincial Center for Disease Control and Prevention, 6 Zhuodaoquan North Road, Hongshan District, Wuhan, 430079 China; 3 Global Health Institute, Wuhan University, 115 Donghu Road, Wuchang District, Wuhan City, 430071 China

**Keywords:** Air pollution, Mortality, Effect modifiers, Susceptible subpopulation

## Abstract

**Background:**

Ambient air pollution has posed negative effects on human health. Individual-level factors may modify this effect, but previous studies have controversial conclusions, and evidence is lacking especially in developing countries. This study aims to examine the modifying effects of sex, age, and education level of individuals on the associated between daily mortality and air pollutants, including particulate matter < 10 μm in aerodynamic diameter (PM_10_), sulfur dioxide (SO_2_), and nitrogen dioxide (NO_2_).

**Methods:**

Time-series analysis was conducted to investigate the acute effects of the air pollution on daily mortality from January 2002 to December 2010 in Wuhan, China. Generalized Additive Models (GAM) were used to examine the association stratified by sex for non-accidental, cardiovascular, and respiratory mortality. For non-accidental mortality, stratified analysis was also conducted by age and educational level.

**Results:**

Outdoor air pollution was associated with daily non-accidental and cardiovascular mortality. An increase of 10 μg/m^3^ in a 2-day average concentration of PM_10_, SO_2_, and NO_2_ was corresponding to the increase in non-accidental mortality of 0.29% (95%CI: 0.06–0.53%), 1.22% (95%CI: 0.77–1.67%) and 1.60% (95%CI: 1.00–2.19%), respectively. The effects of air pollution were faster in females than males. The magnitude of the estimates was higher for females with low education, aged 65–75 years for PM_10_ and < 65 years for SO_2_. To be more specific, we observed that per 10 μg/m^3^ increase in SO_2_ was association with increases in non-accidental mortality of 2.03% (95%CI: 1.38–2.67) for all females and 3.10% (95%CI: 2.05–4.16) for females with low education.

**Conclusion:**

Females and people with low-education are more susceptible to the effect of air pollution, which would provide a sound scientific basis for determination of air pollution standards.

## Background

Numerous epidemiological studies have shown exposure to air pollution closely associated with several adverse health outcomes, such as increased total mortality, cause-specific mortality, hospitalizations, and morbidity of asthma and lung cancer [1–5]. Such effect could be modified by individual-level factors including sex, age, and socioeconomic status (SES) [6–13]. These findings indicate that certain populations may be more vulnerable and susceptible to air pollution than others, but the conclusions are controversial [6]. Thus, more evidence of those modifiers could provide a solid scientific basis for public policy determination of prevention, and benefit risk assessment and air pollution standard in China.

Sociodemographic characteristics, like sex, age and SES have been identified as important effect modification in the associations between air pollution and mortality. Females were found more susceptible to the effects of air pollution on total mortality than males in some studies [11, 13, 14], while no evidence was found in others. Although there are inconsistent findings, the populations with specific age ranges were found to have higher risks than others [7, 11]. Researchers assess SES varies, such as education, occupation, family income savings, etc. [15]. Some studies found that people with lower SES may bear disproportionate burdens of air pollution on their health status, [16–19]; however, the precise modification effects of SES remains rather unclear [6]. Given that the differences in sociodemographic factors, and medical and environmental conditions vary in areas, the modifiers of environmental exposure and vulnerable populations could be quite various in different regions [11]. Nevertheless, few studies have been previously conducted in China, where ambient air pollution has been regarded as the fourth leading risk factor for disability-adjusted life-years (DALYs) [20].

This study analyzed individual mortality data and daily air pollutant concentration data, including particulate matter < 10 μm in aerodynamic diameter (PM_10_), sulfur dioxide (SO_2_), and nitrogen dioxide (NO_2_), to assess the modifying effects of sex, age, and education level of individuals on the associations between air pollution and daily mortality. Findings of this study would deepen our understanding of how various groups of populations may be differently influenced by the harm of air pollution, help amendment to the laws on prevention of air pollution, and inform governmental policy-makers of effective ways to conduct interventions.

## Methods

### Study population and data sources

**Fig. 1:**
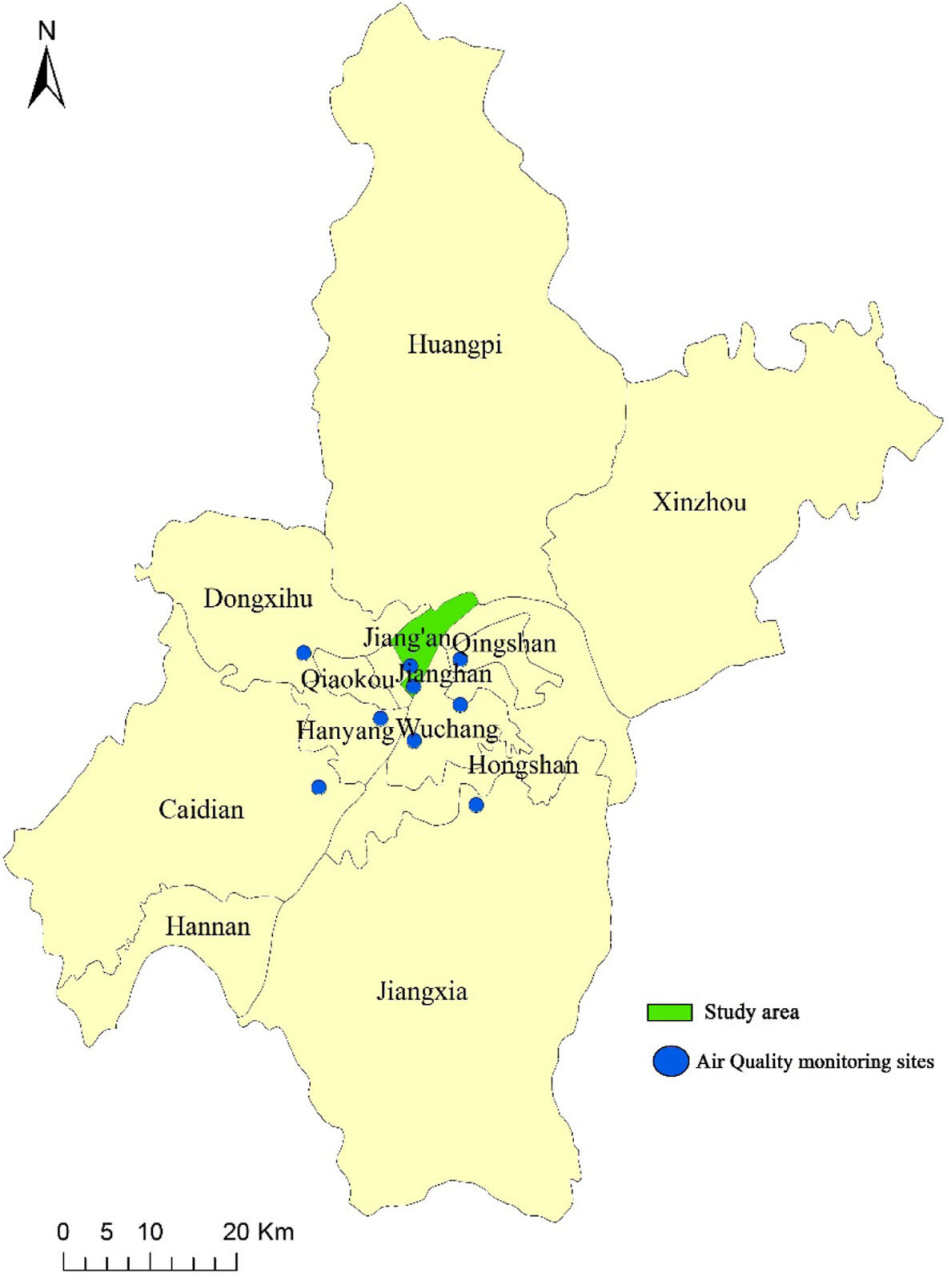
District map of Wuhan with monitoring station locations. Jiang’an District were included in the present study

Residents’ mortality data from January 1st 2002 to December 31st 2010 were obtained from the Centre for Disease Control and Prevention (CDC) of Jiang’an District in Wuhan, China. Wuhan is the capital of Hubei province in central China (Fig. 1), with a total resident population of about 10,220,000 and 8,220,493 registered residents among the total at the end of 2013. Jiang’an, with a total resident population of 926,800 and among which 700,179 registered residents, is considered as the political, economic, cultural and financial center of Wuhan. In addition to higher population density than average in Wuhan (14,427/km^2^ *v.s.* 1203/km^2^), Jiang’an also includes two of nine fixed-site air pollution monitoring stations (Fig. 1), which allows more accurate measurement of individuals’ exposure to air pollution [21].

During the study period, a total of 36,600 registered non-accidental deaths in Jiang’an District were collected in the dataset. The causes of death during the year of 2002 and 2003–2010 were coded based on the International Classification of Diseases, 9th revision (ICD-9) and 10th revision (ICD-10), respectively. All causes excluding accidental deaths (ICD-9 code < 800; ICD-10 code A00-R99), cardiovascular diseases (ICD-9 code 390–459; ICD-10 code I00-I99), and respiratory diseases (ICD-9 code 460–519; ICD-10 code J00-J99) were separately extracted. Individual-level covariates in the mortality data included age, sex, and educational level as an indicator of an individual’s SES. Educational level was classified as low-education (illiterate and primary school) and high-education (middle school and above).

Daily air pollutant data were obtained from the Wuhan Environmental Monitoring Center, including PM_10,_ SO_2_, and NO_2_. The daily concentrations were averaged from the readings of two monitoring stations in the study area. We collected daily meteorological data on temperature and relative humidity from China Meteorological Data Sharing Service System (http://data.cma.cn/en).

### Statistical analysis

Poisson Generalized Additive Models (GAMs) were employed to explore the associations between air pollutants and daily mortality. First, the basic models were built for various mortality outcomes excluding the air pollution variables. The partial autocorrelation function was used to guide the selection of degrees of freedom (df) for the time trend. There are two criteria for selecting the best-fitting: the absolute value of the partial autocorrelation < 0.1 for all 30 days and the smallest sum of the absolute partial autocorrelation values over a 30-day lag period [13, 22]. We used 3 df for temperature and humidity to control their effects on mortality [23]. In our study, 5, 5, and 4 df per year for time trend were used in our basic models for total, cardiovascular, and respiratory mortality, respectively. After the basic models were established, we introduced the pollutant variables and analyzed their effects on mortality outcomes. The final model was as follow:

$$ \log \left[E\left({y}_t\right)\right]=\beta {Z}_t+s\left( time,{df}_1\right)+ as. factor(dow)+\mathrm{a}s. factor(holiday)+s\left( temperature,3\right)+s\left( humidity,3\right)+ intercept $$

where *E*(*y*_*t*_) is the expected number of deaths at day *t*; *Z*_*t*_ is the concentration of air pollutants at day t; *β* is the log-relative rate of mortality associated with a unit increase of air pollutants; s () indicates the smoother based on penalized spline method; df_1_ is the degrees of freedom for controlling season and long-term trend; dow and holiday are dummy variables for day of week and holiday, respectively; 3 df for temperature and humidity.

Stratified analyses by sex were separately conducted for non-accidental, cardiovascular and respiratory mortality. We also examined the associations stratified by age, and education for non-accidental mortality. We tested the statistical significance of differences between effect modifier (e.g., the difference between female and male) by calculating the 95% confidence interval (CI) as:

$$ \left({\widehat{Q}}_1-{\widehat{Q}}_2\right)\pm 1.96\sqrt{S{{\widehat{E}}_1}^2+S{{\widehat{E}}_2}^2} $$

where ${\widehat{Q}}_1$ and ${\widehat{Q}}_2$ are the estimates for the two categories, $\mathrm{S}{\widehat{E}}_1$ and $\mathrm{S}{\widehat{E}}_1$ are their respective standard errors [12].

We also examined the lag effects between air pollution and various mortality outcomes with single-day lag models (lag0 to lag7) and multi-day lag models (lag01 to lag07). For example, lag0 denotes the concentration of air pollutants on the present day while lag1 indicated the previous; lag01 in cumulative exposure models denotes the 2-day moving average concentration of air pollutants concentrations on the present day and previous day.

Sensitive analyses were conducted in three ways: a) checking the impact of df selection on the effect size estimate of three pollutants; b) using indicator variables for days with the highest 1% and lowest 1% values of temperature, and for days with the highest 1% values of humidity; c) using air pollution data from one of the two monitoring stations to address possible exposure misclassification.

All analyses were conducted in R software (version 3.1.3), using mgcv packages. The percent changes and 95% confidence intervals (95% CIs) in daily overall (non-accidental) and cause-specific mortality for each 10 μg/m^3^ increase in the concentration of each pollutant were reported.

## Results

Among 36,600 registered non-accidental deaths, the elder (≥ 65 years) and females accounted for 74.7% and 44.6%, respectively. For non-accidental deaths, the age structures of different gender are similar, with > 70 age group having the highest proportion, males accounting for 43.3% and females 58.0%. The total number of deaths among males in the 65–70 age group was the lowest (23.4%), and in females was < 65 age groups (19.3%). The non-accidental deaths with low educational level between genders varies greatly, male accounting 59.9%, while female only 36.3%. During the study period (2002–2010), the average daily cardiovascular deaths were 5.15, and the average daily respiratory deaths were 1.10. The average daily concentration was 118.65 μg/m^3^ for PM_10_, 49.26 μg/m^3^ for SO_2_, and 58.32 μg/m^3^ for NO_2_. The mean temperature and relative humidity were 17.88 °C and 71.79%, respectively (Table 1).

**Table 1:** Summary statistics of daily deaths, air pollution concentrations and atmosphere variables in Wuhan, China, from 2002 to 2010

variables	Mean ± SD	min	P25	P50	P75	max
Daily deaths						
Deaths causes						
Total non-accidental	11.13 ± 3.98	1.00	8.00	11.00	14.00	34.00
Cardiovascular	5.18 ± 2.67	0.00	3.00	5.00	7.00	23.00
Respiratory	1.10 ± 1.17	0.00	0.00	1.00	2.00	10.00
Gender						
Female	6.17 ± 2.76	0.00	4.00	6.00	8.00	18.00
Male	4.96 ± 2.46	0.00	3.00	5.00	6.00	21.00
Age						
< 65 years	2.83 ± 1.72	0.00	2.00	3.00	4.00	10.00
65~ 75 years	2.68 ± 1.75	0.00	1.00	2.00	4.00	16.00
≥ 75 years	5.63 ± 2.80	0.00	4.00	5.00	7.00	18.00
Weather						
Relative humidity (%)	71.79 ± 12.61	21.00	63.00	73.00	81.00	98.00
Temperature (°C)	17.88 ± 9.35	−2.70	9.70	19.00	25.90	35.80
Air pollutants concentration (μg/m^3^)						
PM_10_	118.65 ± 62.67	10.50	72.00	108.00	152.00	600.00
SO_2_	49.26 ± 33.60	1.00	25.00	41.00	65.50	260.50
NO_2_	58.32 ± 25.37	12.00	39.20	52.80	73.60	288.00

Concentrations of air pollutants were relatively highly correlated with each other, with Spearman’s *r* ranging from 0.62 (between PM_10_ and SO_2_), to 0.73 (between PM_10_ and NO_2_). PM_10_, SO_2_, and NO_2_ concentrations were all negatively correlated with temperature (Table 2).

**Table 2:** Spearman correlation analysis between air pollutants and atmosphere variables in 2002–2010

	PM_10_	SO_2_	NO_2_	Temperature
SO2	0.62*			
NO2	0.73*	0.69*		
Temperature	−0.24*	−0.31*	−0.21*	
Relative humidity	−0.21*	− 0.28*	− 0.24*	− 0.17*

^*^p < 0.001

Given that most statistically significant associations were observed for lag0 and lag1 with single-day lag models, only the percent change in daily overall (non-accidental) and cause-specific mortality associated with 10 μg/m^3^ increase in air pollutants for lag0, lag1 and lag01 were reported (Table 3). For example, with an increase of 10 μg/m^3^ in 2-day average concentration of PM_10_, SO_2_, and NO_2,_ daily non-accidental mortality increased by 0.29% (95% CI = [0.06–0.53]), 1.22% (95% CI = [0.77–1.67]), and 1.60% (95% CI = [1.00–2.19]) respectively; daily cardiovascular mortality increased by 0.51% (95% CI = [0.18–0.83]), 1.32% (95% CI = [0.69–1.95]), and 2.22% (95% CI = [1.37–3.07]), respectively; and daily respiratory mortality increased by 0.67% (95% CI = [0–1.34]), 1.70 (95% CI = [0.41–3.00]), and 1.56% (95% CI = [− 0.25–3.41]), respectively.

**Table 3:** Percent increase (mean and 95% confidence interval) in daily cause-specific mortality associated with a 10-μg/m^3^ increase in air pollutants concentrations by sex at different lag days

	PM_10_		SO_2_		NO_2_	
Non-accidental						
lag0	0.23	0.02–0.44	0.93	0.54–1.32	1.13	0.63–1.64
lag1	0.22	0.02–0.42	0.80	0.42–1.18	1.13	0.62–1.63
lag01	0.29	0.06–0.53	1.22	0.77–1.67	1.60	1.00–2.19
Male						
lag0	0.17	−0.1-0.44	0.35^a^	−0.17-0.86	0.93	0.27–1.60
lag1	0.34	0.08–0.60	0.60	0.11–1.10	1.29	0.63–1.95
lag01	0.32	0.03–0.62	0.65^a^	0.06–1.24	1.54	0.77–2.31
Female						
lag0	0.34	0.04–0.65	1.74^a^	1.18–2.30	1.44	0.71–2.18
lag1	0.06	−0.24-0.36	1.17	0.62–1.72	0.98	0.24–1.72
lag01	0.25	−0.10-0.59	2.03^a^	1.38–2.67	1.69	0.83–2.56
Cardiovascular						
lag0	0.41	0.11–0.70	0.96	0.42–1.51	1.52	0.80–2.24
lag1	0.40	0.12–0.69	0.97	0.44–1.50	1.69	0.97–2.42
lag01	0.51	0.18–0.83	1.32	0.69–1.95	2.22	1.37–3.07
Male						
lag0	0.48	0.09–0.88	0.23^a^	−0.53-0.99	1.44	0.47–2.43
lag1	0.69	0.31–1.08	0.89	0.16–1.62	2.05	1.07–3.03
lag01	0.76	0.32–1.20	0.77^a^	−0.09-1.64	2.43	1.29–3.57
Female						
lag0	0.50	0.08–0.92	1.91^a^	1.15-2.68	1.91	0.89–2.94
lag1	0.23	−0.18-0.65	1.31	0.55–2.06	1.68	0.65–2.72
lag01	0.46	−0.01-0.92	2.19^a^	1.31–3.08	2.47	1.27–3.68
Respiratory						
lag0	0.48	−0.13-1.09	1.17	0.06–2.30	0.99	−0.54-2.55
lag1	0.59	0.00–1.19	1.36	0.27–2.46	1.38	−0.17-2.95
lag01	0.67	0.00–1.34	1.70	0.41–3.00	1.56	−0.25-3.41
Male						
lag0	0.47	−0.29-1.25	0.62	−0.79-2.05	−0.02^a^	−1.96-1.95
lag1	1.08	0.34–1.81	0.99	−0.39-2.39	1.41	−0.54-3.39
lag01	0.99	0.15–1.83	1.06	−0.56-2.71	0.85	−1.41-3.17
Female						
lag0	0.86	−0.09-1.83	2.65	0.90–4.44	3.34^a^	0.94–5.80
lag1	0.17	−0.78-1.13	2.53	0.82–4.28	2.18	−0.26-4.68
lag01	0.62	−0.45-1.69	3.54	1.52–5.60	3.84	0.99–6.78

^a^there were significant difference between males and females

Effect estimates of air pollutants on daily all-type mortality involved in our study also varied by gender (Table 3). Significant differences were observed between males and females in non-accidental and cardiovascular mortality for SO_2_, and in respiratory mortality for NO_2_. Observed effect estimates of SO_2_ at lag01 were approximately 5–6 times higher among females than males. The effect estimates of SO_2_ on non-accidental were 2.03 (95%CI = [1.38–2.67]) for females and 0.65 (95%CI = [0.06–1.24]) for males; it was 3 times higher on cardiovascular mortality for females and males, and the effect estimates were 2.19 (95%CI = [1.31–3.08]) and 0.77 (95%CI = [− 0.09–1.64]), respectively. Effect estimates of NO_2_ at lag01 were approximately 4 times greater among females in respiratory mortality and the effect estimates were 0.85 (95%CI = [− 1.41–3.17]) and 3.84 (95%CI = [0.99–6.78]), respectively.

**Fig. 2:**
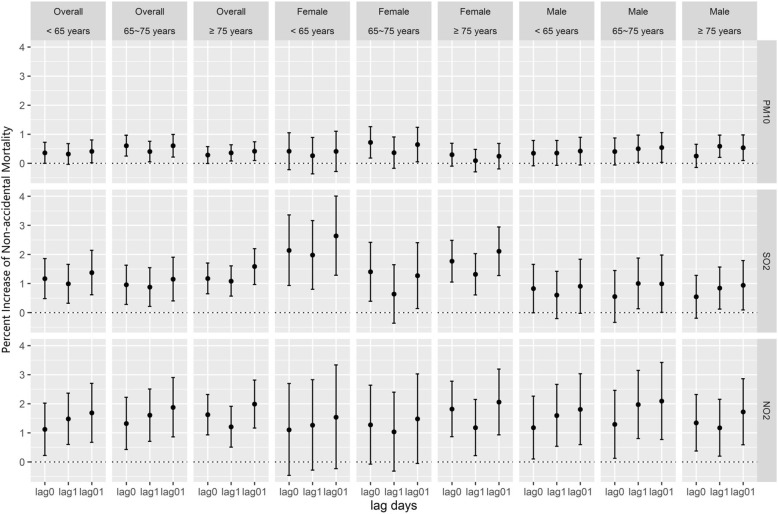
Effect estimates of age categories and age categories for each sex on non-accidental mortality at lag0, lag1, and lag01 for three pollutants

In addition to gender, age is also considered as an effect modifier in association between air pollution and mortality. So, we estimated age-specific and sex-specific effects of three air pollutants on non-accidental mortality (Fig. 2). Effect estimate of PM_10_ in people aged 65–75 years was slightly higher than it in other age categories. When being stratified by sex, the confidence intervals became wider and some of them became not statistically significant anymore, and the highest estimate occurred in females aged 65–75 years. In the case of SO_2_ and NO_2,_ effect estimates were similar and significant among all age subgroups. The estimates were higher in females compared with males for SO_2_, especially in age group < 65 years.

**Fig. 3:**
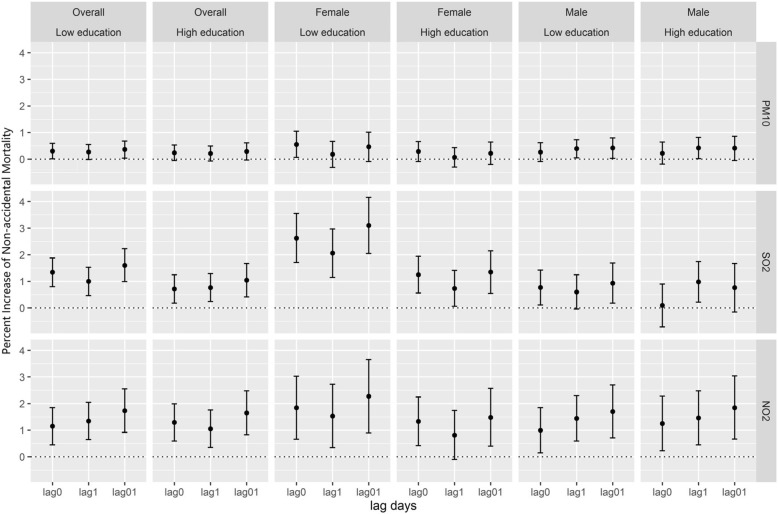
Effect estimates of education categories and education categories for each sex on non-accidental mortality at lag0, lag1, and lag01 for three pollutants

Our finding showed that the low education group had higher effect estimates than the high education group for PM_10_ and SO_2_, but the differences were not significant (Fig. 3). After stratification by sex, the highest estimates were consistently observed in females of low education group. The effect estimates were 0.46% (95%CI = − 0.09–1.01) for PM_10_, 3.10% (95%CI = 2.05–4.16) for SO_2_ and 2.27% (95%CI = 0.90–3.65) for NO_2_ at lag01, respectively.

## Discussion

We explored the influences of three air pollutants on health effect on different populations in Wuhan, China. Our findings shed light on significant associations between air pollution and daily non-accidental mortality modified by individual factors, especially by gender. Adverse effects of short-term exposure to air pollution generally occurred faster among females than males. Specifically, effects of SO_2_ on non-accidental, cardiovascular, and respiratory mortality and effects of NO_2_ on respiratory mortality were stronger among females than males. Lower education might intensify the adverse effect of air pollution.

In our study, the health effect estimates of three air pollutants were close at lag0 and lag1 on all research mortality. Previous related studies in Asia suggested the highest estimates of a single day occurred on the present day (lag 0) or previous day (lag 1) for nature mortality, whereas on lag 2 for respiratory mortality [24–26]. Stratified by sex, the acute effect of air pollutants (PM_10_, SO_2_, and NO_2_) was observed to be faster in females than in males, for the highest effects occurred on current day in females and on the previous day in males (Table 3). The results are similar even after stratified by age and education for both genders (Fig. 2; Fig. 3). It suggested that females were more vulnerable to air pollution, not only reflected in the effect estimates, but also in the lag times. More specifically, for both genders, per 10 μg/m^3^ increase of PM_10_ was associated with an increase of 0.23%, 0.22% at lag 0 and lag 1 in non-accidental mortality, respectively. The corresponding values are 0.17% and 0.34% for males, whereas 0.34% and 0.06% for females at lag 0 and lag 1. The results indicated that different single-day lag effects varied by sex might underestimate the health effects of air pollution in single-day lag models [27].

Females had higher risk estimates of SO_2_ on non-accidental, cardiovascular, and respiratory mortality than males. This is consistent with the majority of previous findings that a slightly higher effect of SO_2_ was found in females than males on the overall mortality [13, 28]. SO_2_ is an acknowledged respiratory irritant and could lead to bronchoconstriction, which would increase airway resistance. SO_2_ partly converts through chemical reactions to sulfate particles, which is an important component of PM_2.5_ [29]. The concentration of SO_4_^2−^ in the PM_2.5_ was found positively associated with daily mortality and hospitalization [30–32]. Moreover, deposition of particles in the lung varies by gender, with greater lung deposition of fine particulate matter in a range of particle sizes for females [33].

Our study also found higher effects of NO_2_ on respiratory mortality in females than in males, with per 10 μg/m^3^ increase associated with a 3.84% increase in respiratory mortality for females. Both short-term and long-term exposure to NO_2_ could affect human health [3, 34]. NO_2_ is a gaseous pollutant which contributes to respiratory inflammation infections and symptoms [35]. People suffering from respiratory diseases, like asthma, are very sensitive to NO_2_ at high concentrations [36]. The females with respiratory diseases are more likely to be influenced by NO_2_, who have greater airway reactivity than males [37].

Physiological differences between genders might be a possible explanation for different susceptibilities. Compared to males, females have relatively smaller lung size, narrower airways and greater airway reactivity [37], and thus have more deposition of particles deposition [38] and more gas absorption [39]. Additionally, smoking rate is lower among females than males in China, while it was pointed that non-smokers could be more susceptible to air pollution than smokers [40].

Apart from the physiological differences, social differences between genders (e.g., self-representation, socially derived activities and roles) lead to various activity patterns and locations, thereby shaping exposure distribution [41]. Furthermore, these differences influence the health effect assessment in air pollution epidemiology studies. Nevertheless, the true factors between males and females could not be fully identified by time-series analysis. Thus, more accuracy exposure assessment and better study design should be done for gender analysis in air pollution epidemiology.

In our study, people aged 65–75 years were at a slightly higher risk of PM_10_ for non-accidental mortality than other age groups, which was consistent with a study in Korea [11]. Also, a systematic review and meta-analysis of susceptibility to health risks associated with particulate matter reported evidence that the elderly (≥65 years) experience higher risk [6]. However, high risk population was identified of much older age groups in other studies. Zeka et al. found that those older than 75 years were at higher risk than younger age groups, and the difference was statistically significant in 20 cities of Units States. A study in Canada reported effect modification by age, and the age group of at least 85 years were at highest risk [7]. Regarding our finding, one possible explanation is healthy survivor effect [11]. Many ill persons have already died when individuals came to their 70s, so the surviving older population was not that susceptible and healthy enough to reach later ages.

As an indicator of SES, education level has often been used in time-series studies [12]. In present study, we examined susceptibilities to air pollution stratified by education and found higher effects were observed in the lower education category for non-accidental mortality, though the differences were insignificant. Low SES may increase vulnerability to air pollution in several pathways, such as limited access to health care, poor nutrition, higher possibilities to live in poor housing conditions without air conditioning and living closer to busy roadways [11]. Sex is also commonly used as a predictor of SES [12]. Some studies pointed out that women’s lower average s education may confound gender and SES in China [13]. In our study, extremely high effect estimate (3.10%, 95%CI = 2.05–4.16) was found for females receiving low education on non-accidental mortality with per 10 μg/m^3^ increase in SO_2_. SES is also related to many other factors such as housing type, the air conditioning system at the residence, and history of socioeconomic conditions [11]. Thus, further studies are needed to investigate the overall effects of SES as effect modifiers of air pollution-mortality associations.

Big cities in China have a large proportion of internal migrants, which can affect the precision of the mortality data. Besides, a large geographical study area with relatively heterogeneous populations in time-series studies may increase the exposure misclassification [25, 42–44]. All of above issues might lead to bias of risk estimates of air pollution. Therefore, in this study, we focused on a relatively small area and a stable, homogeneous population to reduce the bias.

The limitations of our analysis should be taken into consideration. Similar with previous air pollution mortality studies [45, 46], we used average concentration of air pollutants from two monitor stations to represent the actual individual exposures. Exposure measurement error was regarded as a major limitation in our study, due to the nature of the ecological studies. The personal mobility and time-activity patterns may also affect the exposure measurement, especially in a relatively small study area in our study. However, given that the study population was permanent residents, most of whom living and working in the same area, the misclassification of exposure was acceptable. Besides, we used a relatively long study period, so the small chance of deaths due to cause-specific diseases may limit the ability to detect tenuous differences in potential effect modifiers but would not substantially affect our main findings.

## Conclusion

In summary, we found that ambient air pollution was associated with daily non-accidental and cardiovascular mortality in Wuhan during 2002 to 2010. Furthermore, our results suggested that females and people with low-education were more vulnerable to air pollution. These findings provide information on vulnerable subpopulations, which would help make protective measures and air pollutant standard in China.

## Abbreviations

CI: Confidential interval
GAM: Poisson Generalized Additive Models
ICD-10: The 10th Revision of the International Classification of Disease
NO_2_: Nitrogen dioxide
PM_10_: Particulate matter < 10 μm in aerodynamic diameter
SO_2_: Sulfur dioxide
